# A Multimodal In‐Situ Liquid Phase TEM and Electrochemical Study on Boric Acid as Electrolyte Additive for Alkaline Zinc‐Air Batteries

**DOI:** 10.1002/smtd.71069

**Published:** 2026-10-12

**Authors:** Sarmila Dutta, Yasin Emre Durmus, Eunmi Im, Hongyu Sun, Hugo Pérez Garza, Shibabrata Basak, Hans Kungl, Hermann Tempel, Rüdiger‐A. Eichel

**Affiliations:** ^1^ Institute of Energy Technologies – Fundamental Electrochemistry (IET‐1) Forschungszentrum Jülich GmbH Jülich Germany; ^2^ Institute of Physical Chemistry RWTH Aachen University Aachen Germany; ^3^ DENSsolutions B.V. Delft Netherlands; ^4^ Faculty of Mechanical Engineering RWTH Aachen University Aachen Germany

**Keywords:** boric acid, electrolyte additive, in‐situ liquid phase TEM, multimodal study, Zn‐air battery

## Abstract

Rechargeable aqueous zinc‐air batteries are critically hindered by dendrite formation, parasitic hydrogen evolution (HER), and corrosion at the zinc anode. Here, we employ a multimodal characterization strategy, bridging nanoscale visualization with macroscale information, to understand the effects of boric acid (BA) as an additive in alkaline media. In‐situ liquid phase TEM (LPTEM) observations demonstrate that BA additive suppresses HER and promotes a uniform, compact plating with highly reversible stripping. This contrasts sharply with the base electrolyte, which shows vigorous bubbling, nonuniform plating, and incomplete stripping. This nanoscale mechanism translates to the macroscale, where BA induces a transformation from dendritic structures to the desirable, compact “boulder‐like” deposit. Macroscale electrochemical analysis quantifies this effect, revealing that 130 mM BA suppresses the corrosion current density by an order of magnitude significantly inhibiting HER. Further investigations combining XRD, ICP‐OES suggest that BA is likely to exist as the tetrahydoxyborate anion, which functions as a non‐consumed, non‐incorporated surface‐active agent. Finally the cycling of ZnO‐PTFE composite anode in BA containing electrolyte over 75 cycles at a current density of 120 mAh/g_Zn_ at 30% DOD with 100% columbic efficiency shows the promise of BA as a practical electrolyte additive.

## Graphical abstract

This study establishes a multimodal correlative workflow to bridge disparate analytical scales in battery research. By linking dynamic in‐situ liquid‐phase TEM at the nanoscale with microscale morphological analysis and macroscale electrochemistry, a comprehensive mechanistic evaluation of electrolyte additives is achieved. Applied to boric acid, this cross‐scale methodology demonstrates how localized nanoscale phenomena dictate macroscopic device performance.

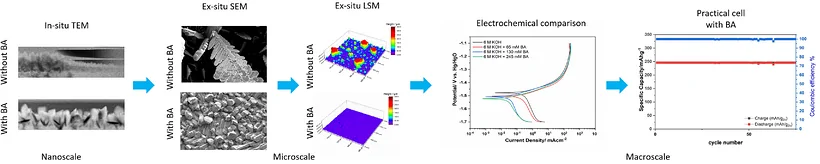

## 1 Introduction

Environmental‐friendly aqueous metal‐air batteries have recently attracted significant interest as a solution for next‐generation electrochemical energy storage, primarily due to their high theoretical energy densities [1]. Among various metal‐air batteries, zinc‐air batteries are particularly encouraging for their low‐cost, competitive energy density, affordability, safety and environmental compatibility [2, 3]. Despite these advantages, secondary zinc‐air batteries face considerable challenges, especially in alkaline electrolytes, which impede their development and commercialization. At the zinc electrode, issues such as passivation, dendrite formation, and the hydrogen evolution reaction (HER) during operation critically hinder performance, reducing discharge capacity, shelf life, and cycling stability [4]. An effective approach to enhance the performance of rechargeable zinc‐air batteries is by using electrolyte additives, which can enhance battery efficiency without requiring significant changes to other components of the system.

Electrolyte additives can operate through several distinct mechanisms to suppress dendrite growth and corrosion [5]. While some additives physically or chemically adsorb onto the metal surface to block active zinc sites [5, 6], others function by altering the solvation sheath or forming complex ions with zinc species in the electrolyte [5, 7, 8]. The specific mechanism is highly dependent on the nature of the additive (e.g., polarity, size) and the chemical environment of the electrolyte [6]. Moreover, certain additives that contain metal ions with a more positive electrode potential than zinc can undergo reduction before zinc plating, resulting in modified surface with optimized current distribution [9]. Various electrolyte additives have been tested in alkaline zinc‐air batteries. For example, Zhu et al. [10] demonstrated that using surfactant additives, particularly with fluorosurfactants such as FC 170, FC 135, as well as the cationic surfactant cetyltrimethylammonium bromide (CTAB), in a 30% KOH electrolyte effectively reduced HER and dendritic growth during zinc plating, Similarly, Dobryszycki et al. [11] reported that the incorporating the organic additive polyethylene glycol into a 6 M KOH electrolyte reduced zinc corrosion. Despite these advancements, the development of alternative and more effective additives remains an active area of research.

On the other hand, boric acid (H_3_BO_3_, BA) is a widely used industrial reagent [12] and has also found applications as an electrolyte additive in various electrochemical systems. Badawy et al. [13] reported that the use of BA in lead‐acid batteries improves the kinetics of PbO_2_/PbSO_4_ couple. Wu et al. [14] found that the addition of BA increase hydrogen and oxygen evolution overpotential in lead‐acid batteries. Additionally, Karwas et al. [15] noticed that the presence of BA accelerates the primary nucleation rate while suppressing secondary nucleation process during electroplating of Ni‐Zn alloy. Gimenez‐Garcia et al. [16] studied the effects of BA on iron electroplating and revealed that its presence leads to improved faradic efficiency, reduce HER and lead to compact deposition morphology. Liu et al. [17] investigated how the addition of BA limits the corrosion in aqueous ZnSO_4_ electrolyte during both immersion and electrochemical processes. They concluded that moderate ligand field interactions could partially replace H_2_O molecules in the solvation sheath of Zn^2+^ ions, forming a stable, water‐deficient solvation sheath that reduces corrosion. The addition of BA also decreased the HER rate and resulted in a dendrite free, uniform surface. Furthermore, they demonstrated that incorporating BA increased the cycle life of Zn||Zn symmetric cells by more than twenty times. However, these findings are specific to neutral or acidic ZnSO_4_ systems. The influence of BA in highly alkaline electrolytes, where the likely active species are tetrahydroxyborate [B(OH)_4_]^−^ and zincate [Zn(OH)_4_]^2^^−^, presents a fundamentally different chemical environment that has not yet been explored.

Electrochemical processes are fundamentally governed by nanoscale phenomena at the electrode–electrolyte interface. Thus, visualizing how an electrolyte additive impacts the plating and HER is essential for mechanistic understanding. Transmission electron microscopy (TEM) is uniquely suited for this, offering the high spatial and temporal resolution required to capture these dynamic processes. The advancement of in‐situ liquid phase TEM (LPTEM) allows researchers to apply an electrochemical stimulus (a bias) directly to an electrode while it is submerged in a liquid electrolyte, enabling direct observation of plating and stripping at the required scale [18, 19, 20]. While various liquid cells exist, MEMS (micro‐electro‐mechanical systems)‐based nanoreactors are particularly effective for applying controlled electrochemical stimuli. However, despite this, apart from the radiolysis too, LPTEM faces significant challenges, including the management of electron beam‐induced unwanted deposition and the handling of gas bubbles, which could alter the actual electrochemical process in aqueous electrolyte systems. In a previous work, we showed incorporating on‐chip electrolyte flow via a pressure‐based pump can overcome beam‐induced unwanted deposition and clear bubbles by continuously replenishing the electrolyte [21]. This makes it feasible to understand the influence of electrolyte additives of an alkaline system after adapting the holder components for alkaline solutions. However, a more fundamental challenge remains: the environment of the MEMS nanoreactor, with its limited field of view, femtoliter volumes, and unique hydrodynamics, makes it difficult to directly extrapolate nanoscale observations to the performance of a bulk battery [22]. A common gap in the literature is the failure to bridge these scales; nanoscale visualizations are often presented without quantitative macroscale validation. To achieve a comprehensive and mechanistically sound understanding, in‐situ TEM observations must be rigorously correlated with macroscale electrochemical investigations.

Recent advancements in in‐situ liquid‐phase TEM (LPTEM) have provided profound insights into the dynamic behavior of zinc anodes. For instance, Dachraoui et al. [23] elegantly utilized LPTEM in neutral 0.1 M ZnSO_4_ to reveal that zinc dendrites fundamentally evolve through the vertical stacking of hexagonal nanosheets and anisotropic branching at high‐energy corners. Huang et al. [24] also employed operando TEM to demonstrate how altering the electrolyte composition in mildly acidic media can regulate this growth; they showed that Mn^2+^ additives precipitate into Mn(OH)_2_ clusters to mechanically homogenize ion flux, whereas CF_3_SO_3_^−^ anions alter the zinc solvation structure to mitigate parasitic hydrogen evolution. However, these LPTEM investigations have predominantly focused on neutral or mildly acidic sulfate and triflate systems. The dynamic interfacial mechanisms in highly alkaline electrolytes which are standard for zinc‐air batteries but notoriously difficult to image in‐situ due to aggressive, vision‐obscuring hydrogen gas evolution remain largely unexplored at the nanoscale. Furthermore, while previous LPTEM studies have identified intrinsic crystallographic pathways and bulk solvation effects, there is a critical need to visualize how surface‐active additives operate in alkaline media and, importantly, to correlate these highly localized nanoscale observations with practical macroscale battery performance.

To bridge the gap between nanoscale mechanisms and macroscale performance, this study employs a correlated, multimodal characterization strategy to explore the influence of BA as an electrolyte additive for alkaline zinc‐air batteries. LPTEM directly visualized the electrochemical processes at the nanoscale, providing unprecedented insight into the additive's function. These nanoscale observations are then systematically correlated with macroscale studies to quantify BA's impact on corrosion, plating morphology, and long‐term cycling performance. Through this integrated approach, we determine the optimal BA concentration and elucidate a holistic working mechanism.

## 2 Materials and Methods

Aqueous KOH solutions were prepared by dissolving KOH crystals (≥85% purity, Sigma‐Aldrich) in deionized water obtained from a PURELAB Elga water purification system. Boric acid (BA, Sigma‐Aldrich) was evaluated as an electrolyte additive. For in‐situ LPTEM experiments, a base electrolyte of 1 M KOH containing 30 mM ZnO (Sigma‐Aldrich) was utilized. To test the effect of the electrolyte additive, 20 mM BA was introduced to this base solution. For macroscale electrochemical tests, BA was evaluated at varying concentrations (65, 130, and 245 mM) in 6 M KOH. Specifically for zinc plating experiments, 0.2 M ZnO was added to these KOH electrolytes.

For macroscale electrochemical testing, zinc working electrodes were prepared by sectioning a zinc rod (1.26 cm diameter) into 1.2 mm thick discs using a Struers Accutom‐100 cutting machine. The embedded zinc discs were mechanically polished to a mirror finish using 800‐ and 1200‐grit grinding paper, followed by diamond polishing suspensions (MolR3 and NapR1, DiaPro).

In‐situ LPTEM studies were conducted using a commercially available liquid TEM holder Stream from DENSsolutions B.V. adapting components suitable for alkaline electrolytes in a probe‐corrected TFS Titan microscope operating at 200 kV in scanning transmission electron (STEM) mode. The assembled MEMS‐based nanoreactor with the aligned transparent SiN_x_ window allowed visualization of the plating and stripping processes over a platinum (Pt) microelectrode, while an on‐chip Pt microelectrode serves as a pseudo reference electrode. First, the plating and stripping processes were studied using a base electrolyte (1 M KOH + 30 mM ZnO). Then, the effect of adding BA was investigated by flowing the base electrolyte with 20 mM BA solution on the same assembly after flushing the nanoreactor for 30 mins with the BA containing electrolyte (during this period, the working electrode was held at 0 V to strip any residual Zn deposits). Please note, a 1 M KOH concentration was used for LPTEM experiments, as opposed to the 6 M concentration used in macroscale tests. This dilution was necessary to ensure a stable electrolyte flow within the MEMS nanoreactor, prevent channel clogging, and minimize aggressive beam‐induced artifacts that are prevalent in highly concentrated electrolytes. All the experiments were conducted at a flow rate of 1.3 µL/min using a pressure‐based pump from Fluigent. A critical consideration in quantitative LPTEM is mitigating the influence of electron‐beam irradiation, which can induce water radiolysis, localized pH shifts, and artificial deposition. To ensure the observed interfacial processes were electrochemically driven, a rigorous mitigation and optimization protocol was employed. All imaging was performed in HAADF‐STEM mode at 200 kV, with a beam current of 20pA with a dwell time of 0.33µs. The rastering nature of the STEM probe, combined with a continuous electrolyte flow rate of 1.3 µL/min, actively flushed radiolytic by‐products from the approximately 500 nm thick liquid layer before they could accumulate and alter the local chemistry. To distinguish intrinsic electrochemical behavior from beam‐induced artifacts, an active baseline tuning step was performed prior to cycling. The electrode was monitored at open‐circuit potential (OCP), and imaging parameters were fine‐tuned until a “net‐zero” balance of beam‐induced growth and dissolution was achieved, confirming a stable environment. The absence of beam‐induced artifacts was further validated by the strict dependence of zinc plating and stripping on the applied cathodic and anodic potentials. Finally, because identical imaging and flow parameters were maintained across all experiments, the striking suppression of gas evolution and morphological transformation observed in the BA‐containing electrolyte can be definitively attributed to the intrinsic electrochemical function of the additive.

The ZnO‐PTFE anodes with 2 wt.% Bi_2_O_3_ were prepared following our recent work [25]. In short, anode mixture of ZnO nanoparticles and PTFE were prepared in a weight ratio of 85:15 and with 2 wt.% (with respect to the ZnO weight) Bi_2_O_3_ additive using planetary ball (Fritsch, Pulverisette 5) using iso‐propanol. Then the anode mixtures were fabricated into 1 mm thick anodes, incorporating Ni meshes of 1 cm × 1 cm as current collectors, sandwiched between the anode materials using roll press (MTI) at linear roller speed of 25 mms^−1^ at room temperature. The electrochemical cycling were conducted in a half‐cell configuration, using three‐electrode cells, with the prepared ZnO‐PTFE anodes serving as the anode, a platinum mesh as the counter electrode, and Hg/HgO as the reference electrode. First, to convert zinc oxide to zinc, the prepared anodes were subjected to a constant current of 160 mA g_Zn_^−1^ in 6 M KOH. Following this, galvanostatic cycling were performed at 120 mA g_Zn_^−1^ at 30% depths of discharge (DOD) in a 6 M KOH with 0.2 M ZnO electrolyte with or with 130 mM BA. The upper cutoff potential was set at ‐1.2 V and the lower cutoff potential at ‐1.8 V, with respect to Hg/HgO reference.

The amount of hydrogen gas evolved during galvanostatic charging also followed the same procedure described in our recent study [25]. In short, utilizing anion exchange membrane in a homemade three‐electrode closed cell, Ar gas was flown into the anodic compartment through one port, and the resultant gas was collected through another port into a gas bag. The concentration of hydrogen in the gas bags was measured using a gas chromatograph (Thermo Scientific Trace 1310).

All bulk electrochemical measurements were conducted in three‐electrode beaker cells containing 60 mL electrolyte for plating experiments and 20 mL electrolyte for all other macroscale experiments. Cyclic voltammetry and potentiodynamic polarization experiments utilized the prepared zinc electrode as the anode, a Pt mesh as the counter electrode, and Hg/HgO as the reference electrode. For macro scale zinc plating and ICP‐OES experiments, the Pt mesh was replaced with a zinc counter electrode.

The morphological evolution of the macroscale zinc deposits was visualized using a scanning electron microscope (FEI Quanta FEG 650) operated at an accelerating voltage of 20 kV. Three‐dimensional surface topography was analyzed via laser scanning microscopy (LSM) using an Olympus laser scanning microscope. Crystallographic phases were identified using x‐ray diffraction (XRD, Malvern Panalytical) with a Cu Kα radiation source operated at 55 kV and 40 mA, scanning over a 2θ range of 20° to 90°.

## 3 Results and Discussions

### 3.1 In‐Situ LPTEM: Nanoscale Visualization of the BA Effect

In‐situ LPTEM experiments were performed using a commercial electrochemical TEM holder adapted for KOH based electrolyte uses to visualize the effect of BA at the nanoscale directly. Before discussing the morphological evolution during plating/stripping, it is essential to establish a framework for interpreting the complex in‐situ LPTEM data. Figure 1 schematically points out the main focal points. First, as depicted in Figure 1a, the geometry of the Pt working electrode creates a highly nonuniform electric field—the local current density at the edges of the electrode is approximately two orders of magnitude higher than on the flat top surface. Generally, this concentrated current density at the edges acts as a driving force for accelerated, nonuniform plating and, ultimately, dendrite growth [23]. Therefore, to accurately assess the additive's effectiveness, it is crucial to compare equivalent localized regions: the flat top of the electrode versus the flat top, and the high‐current edges versus the high‐current edges. Furthermore, when interpreting the HAADF‐STEM projection images, it is important to account for Z‐contrast mechanics. Due to the vast difference in atomic number between the platinum microelectrode (*Z* = 78) and the depositing zinc (*Z* = 30), zinc plating on the flat top of the Pt microelectrode generates minimal contrast against the Pt background and the scattering thick liquid electrolyte layer. Conversely, while zinc electrodeposition is strictly confined to the active Pt surface, morphological growth at the electrode boundaries extends laterally. Consequently, in the 2D projection, these peripheral zinc deposits (at the edges of the electrode) extend to solely electron‐transparent SiN_x_ window, yielding striking contrast changes. Accurately tracking localized plating and stripping activity on the top surface therefore requires a careful evaluation of subtle, dynamic contrast shifts. Because static snapshots only capture discrete states, these continuous dynamics are best observed in the corresponding supplementary videos. It should be noted that the simplified schematic in Figure 1a omitted the reference electrode. The optical image in Figure S1 shows the on‐chip electrode configuration.

**FIGURE 1:**
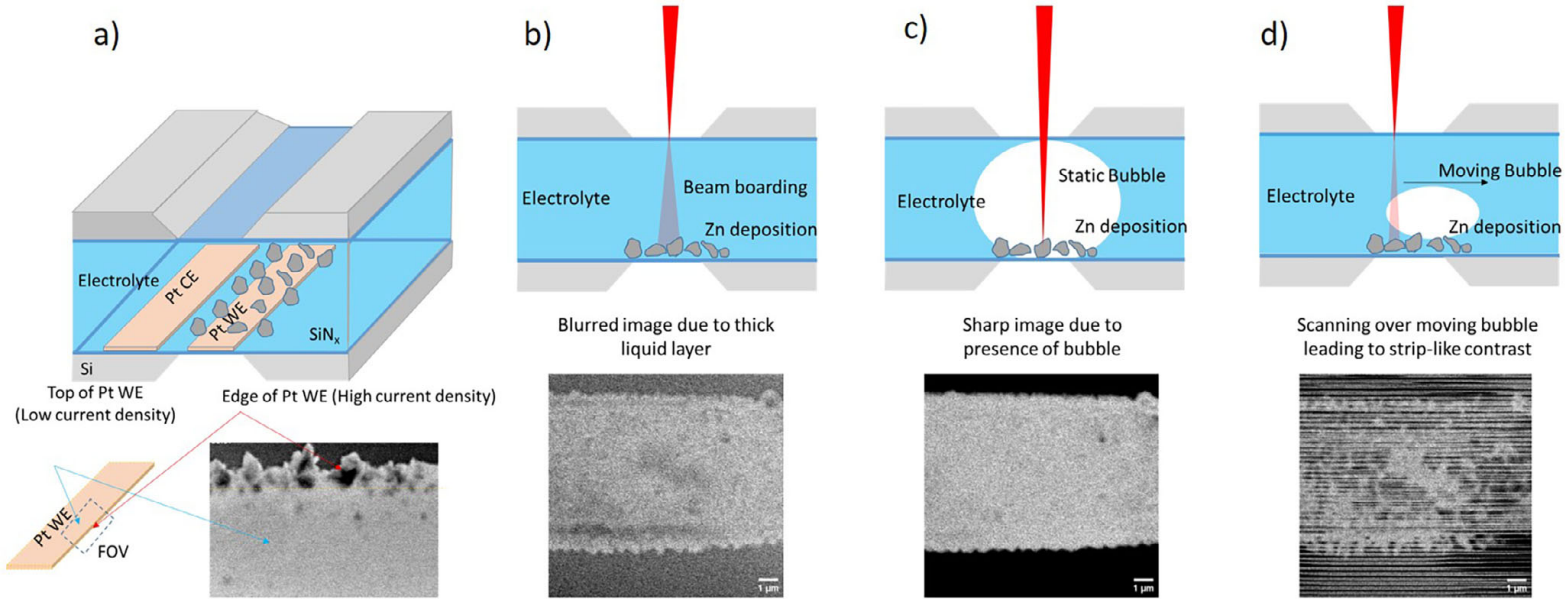
Conceptual representation of the in‐situ LPTEM experimental setup and interpretation guide for dynamic imaging artifacts. (a) 3D schematic of the assembled liquid cell within the MEMS nanoreactor, highlighting the Pt working electrode (WE) and counter electrode (CE). The local current density varies significantly with electrode geometry. (b) Cross‐sectional view depicting imaging through a thick liquid electrolyte layer (no bubbles). Electron beam broadening in the liquid results in a blurred, low‐contrast image. (c) Imaging through a static hydrogen gas bubble. The displaced electrolyte minimizes the local liquid thickness, yielding a sharp, high‐resolution image. (d) Scanning the electron beam over a dynamically moving bubble produces characteristic stripe‐like contrast artifacts in the captured frame.

Furthermore, the image contrast and local plating/stripping observed in these videos are strongly coupled to the dynamic behavior of hydrogen gas bubbles. In regions devoid of bubbles, the relatively thick liquid electrolyte scatters the electron beam, resulting in lower image contrast (Figure 1b). Conversely, when a large, stable gas bubble adheres to the electrode, it displaces the electrolyte and reduces the local liquid thickness, yielding the sharpest, highest‐resolution images of the electrode surface (Figure 1c). However, it is imperative to note that this gas bubble effectively breaks the local electrochemical circuit. Within the footprint of the bubble, Faradaic processes are completely halted—no plating or stripping occurs in these regions regardless of the applied potential. Finally, if bubbles move dynamically across the field of view while the electron beam is rastering, they produce stripe‐like contrast artifacts within the captured frame (Figure 1d).

While Figure 1 illustrates these imaging conditions as distinct, idealized scenarios for clarity, the actual in‐situ environment is highly dynamic. The experimental videos reveal a complex, simultaneous mixture of these states—such as multiple bubbles moving irregularly, small localized stationary gas pockets, or a combination of both, affecting the images as well as local plating/stripping. Based on this interpretive framework, the subsequent experiments evaluate plating and stripping at distinct geometric regions of the Pt microelectrode. Initially the field of view captures both the flat top and the edges of the Pt microelectrode (Figures 2 and 3). These observations demonstrate the general plating, stripping, and bubble dynamics under the simultaneous influence of both low and high local current densities (spanning a difference of two orders of magnitude). Subsequently, the focus shifts to the high‐current‐density edges (Figures S4 and S5) to directly compare how the presence or absence of BA influences the plating and stripping processes. In both cases, electrochemical experiments were first conducted in the base electrolyte (1 M KOH + 30 mM ZnO). Following this, the nanoreactor was flushed with the BA‐containing electrolyte (1 M KOH + 30 mM ZnO + 20 mM BA) for 30 min to equilibrate the system before running identical experiments in the presence of BA. The potential was measured with respect to the on‐chip Pt pseudo‐reference electrode, and a constant electrolyte flow rate of 1.3 µL/min was maintained during the LPTEM experiments.

**FIGURE 2:**
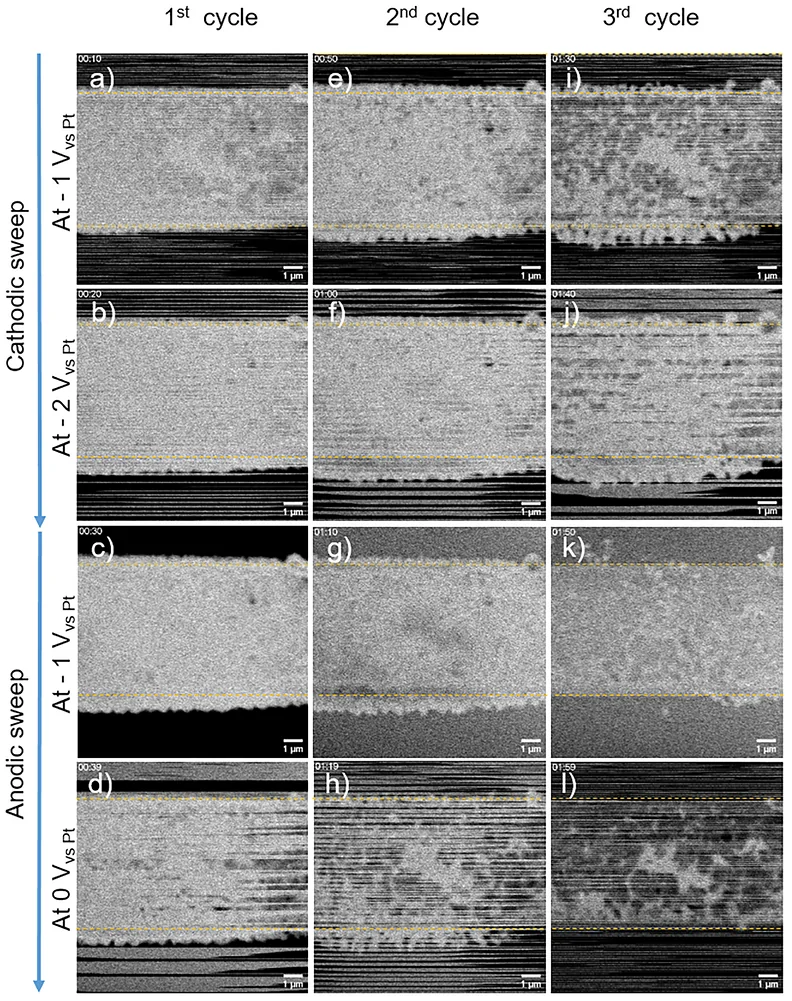
In‐situ HAADF‐STEM snapshots of zinc plating and stripping during the first three CV cycles in the base electrolyte (1 M KOH + 30 mM ZnO). Morphological evolution of the Pt microelectrode over three consecutive cycles at specific applied potentials (Vs Pt): initial cathodic sweep (‐1.0 V), peak deposition (‐2.0 V), initial anodic sweep (‐1.0 V), and post‐stripping residual state (0 V). Yellow dashed lines serve as a guide to distinguish the flat top (low local current density) from the outer edges (high local current density) of the electrode, while timestamps indicate elapsed time (mm:ss).

**FIGURE 3:**
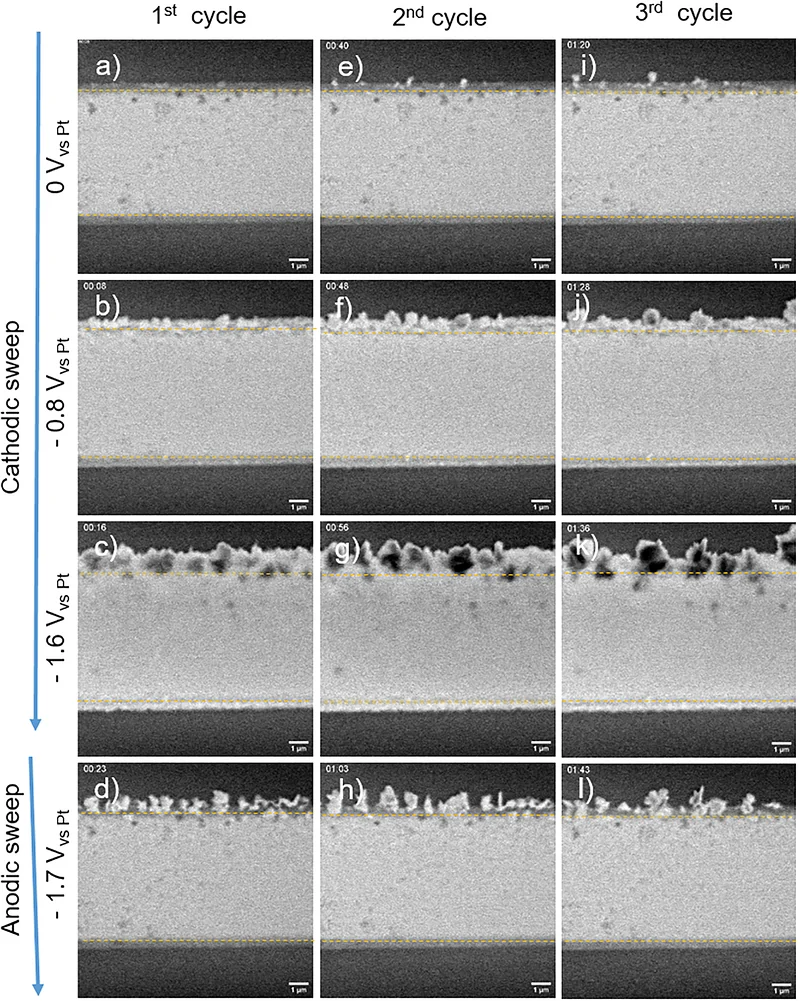
In‐situ HAADF‐STEM snapshots of zinc plating and stripping during the first three CV cycles in the BA‐containing electrolyte (1 M KOH + 30 mM ZnO + 20 mM BA). Morphological evolution of the Pt microelectrode over three consecutive cycles at specific applied potentials (Vs Pt): before deposition (0 V), initial cathodic plating (‐ 0.8 V), near maximum deposition (‐1.6 V), and initial anodic stripping (‐1.7 V). Yellow dashed lines distinguish the flat top (low local current density) from the outer edges (high local current density), while timestamps indicate elapsed time (mm:ss).

**FIGURE 4:**
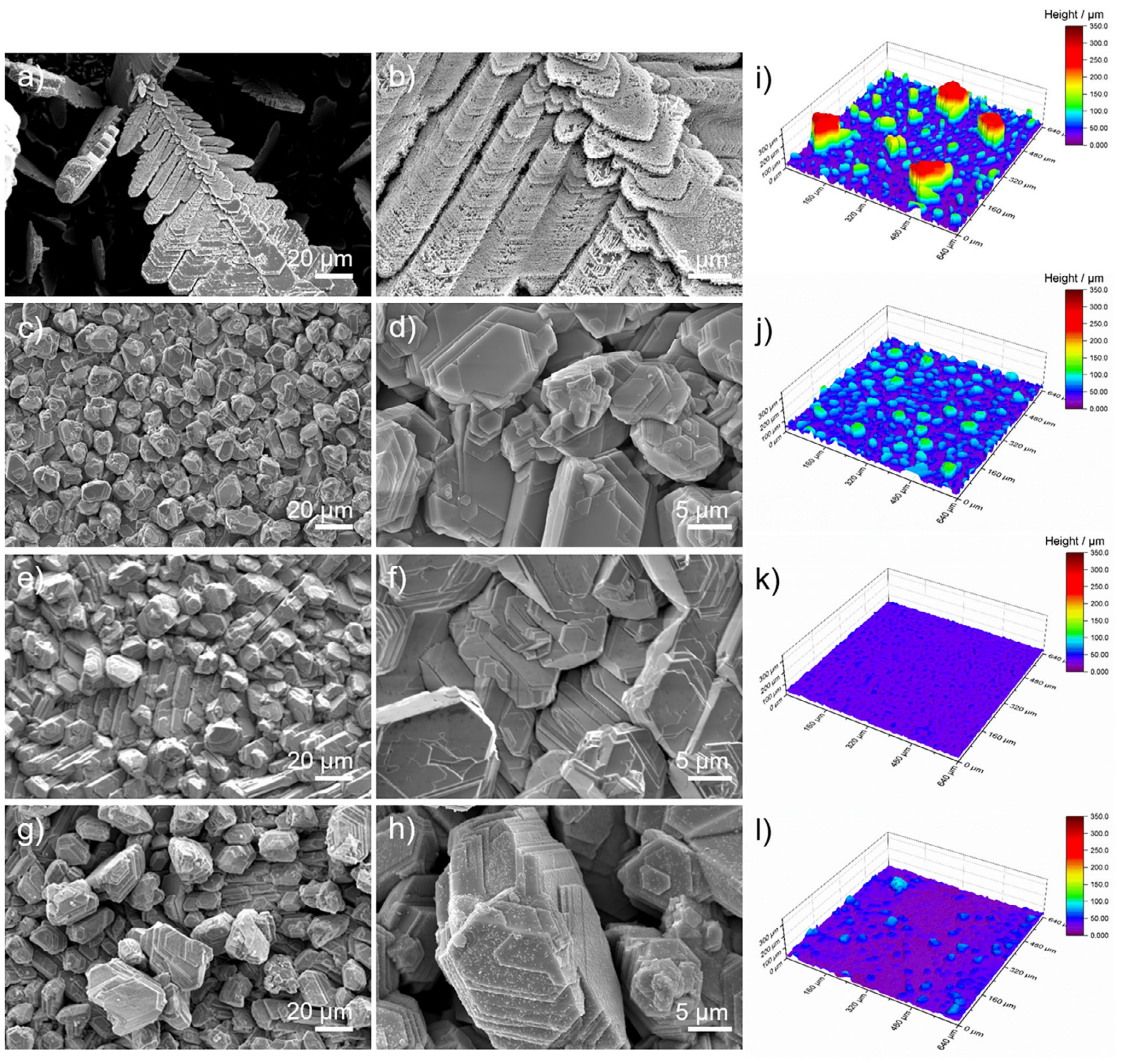
Macroscale plating morphology and uniformity of zinc. (a, b) SEM images of the dendritic, leafy structure formed in the base 6 M KOH + 0.2 M ZnO electrolyte. (c–h) Compact, boulder‐like morphology formed in the presence of (c, d) 65 mM BA, (e, f) 130 mM BA, and (g, h) 245 mM BA. (i–l) Corresponding 3D LSM profiles, which scan the top‐most surface, showing information about the uniformity of the deposition. All platings were conducted at 10 mA/cm^2^ for 1 h.

**FIGURE 5:**
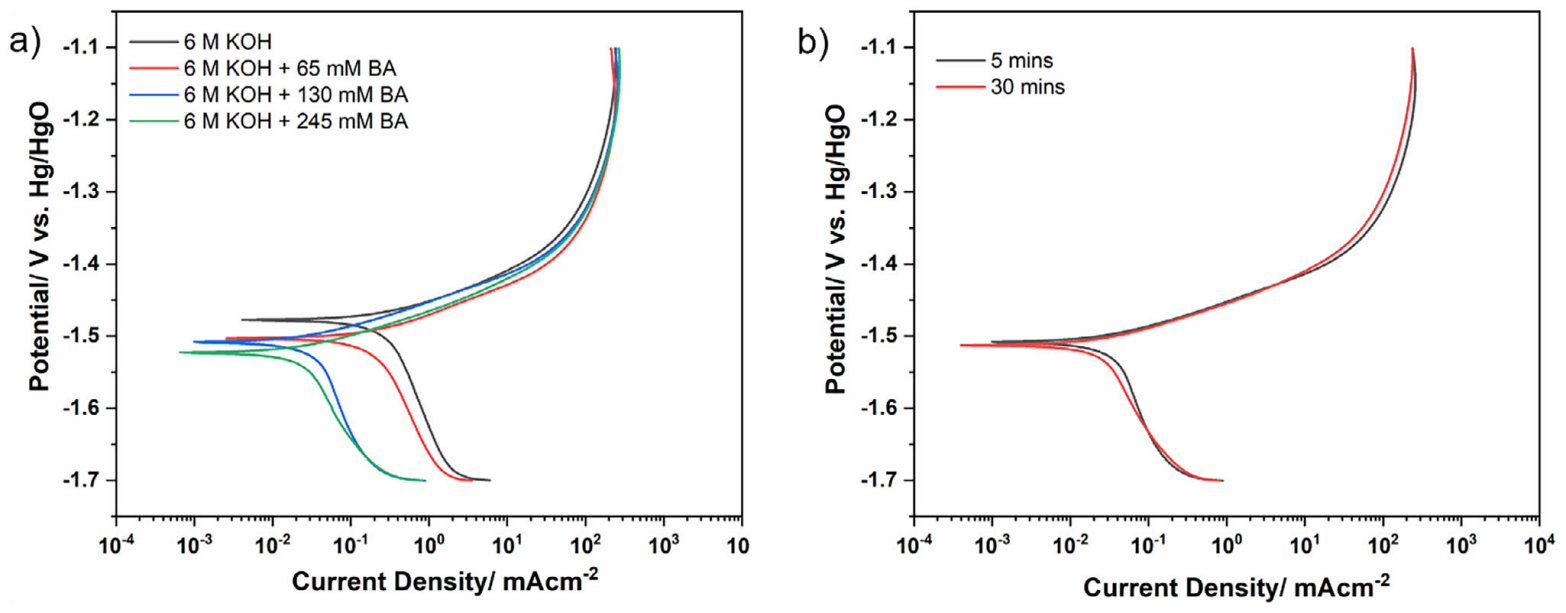
Potentiodynamic polarization studies of zinc in 6 M KOH Electrolyte in absence or presence boric acid (65, 130, and 245 mM) additive. (a) Compares the potentiodynamic polarization curves for different concentration of boric acid; (b) compares the potentiodynamic polarization curves in electrolytes with 130 mM boric acid at different immersion time.

Figure 2 presents the HAADF‐STEM images captured during the first three CV cycles in the base electrolyte. Complete movie of the five CV cycles can be found in Movie 1. Using the framework established in Figure 1, readers should focus on the following aspects to accurately interpret these images: (a) distinguish between the plating and stripping behaviors occurring on the top of the Pt microelectrode versus those at its edges; (b) focus on the subtle shifts in local contrast specifically on the microelectrode's top surface; (c) monitor the evolution of overall image clarity alongside the emergence of dynamic contrast artifacts; and (d) track the progressive build‐up of unstripped material throughout the cycles. By 10 s into the first cycle (Figure 2a), the plating, appearing relatively uniform and dense both on top of the electrode (low current density) and at the edges of the electrode (high current density), has already started. Based on our interpretive framework, the noisy, blurred background is a direct consequence of imaging through a thick liquid layer, while the regions of irregular, stripe‐like contrast (darker or brighter areas with fuzzy edges) is due to the rapid movement of bubbles, providing direct observation of the parasitic HER. By 20 s (Figure 2b), the plated Zn amount continues to grow both on top of the electrode (note the subtle changes in local contrast on top of the Pt microelectrode between Figure 2a and 2b) and at the edge of the electrode. The bubble artifacts persist. By 30 s (Figure 2c), the stripping phase has initiated. This stripe‐like contrast free, sharp, higher resolution image is the result of presence of a large bubble reducing the local electrolyte thickness. A crucial finding is at the end of the first cycle (Figure 2d) significant residual material remains unevenly distributed on top and at the edges the electrode. While incomplete stripping in alkaline media can be attributed to multiple complex factors such as localized passivation or the detachment of dead zinc, the direct visual evidence from the LPTEM videos suggest that the physical presence of insulating gas bubbles is a key contributing factor that locally shields the electrode and hinders uniform electrochemical activity. This residue has a strong influence on the subsequent cycles.

The plating during the second cycle builds on the nonuniform deposits from the first. By 10 s into the second cycle (Figure 2e), the plating is already less uniform than in the previous cycle, likely due to nucleating on the residue sites. The rest of the cycle (Figure 2f–h) progresses largely similar to the first cycle, while the residue at the end of the cycle appears to be more nonuniform than at the end of the first cycle. In the third cycle (Figure 2i–l), the plating becomes more irregular and localized, and the overall deposited amount appears to decrease significantly. This trend continues in cycles 4 and 5 (see Movie S1). This indicates that in the base electrolyte, the parasitic HER and the resulting bubbles disrupt uniform plating and lead to incomplete stripping. This residual zinc also leads to a gradual decrease in the electrochemical efficiency.

To directly determine the effect of the additive, the nanoreactor was flushed with the BA‐containing electrolyte (1 M KOH + 30 mM ZnO + 20 mM BA), and identical CV cycles were visualized at the exact same section of the Pt microelectrode. Figure 3 presents the corresponding HAADF‐STEM images captured during the first three CV cycles in the BA‐containing electrolyte. The initial state of the microelectrode before the CV cycle can be seen in Figure S2. Complete movie of the five CV cycles can be found in Movie 2. At the beginning of the first cycle, before any plating occurs (Figure 3a), the electrode surface is devoid of zinc. By 8 s to the cathodic sweep, the initial plating, both at the top and at the edges of the microelectrode, has already started. (Figure 3b). The completely free of the stripe‐like contrast artifacts, observed in the base electrolyte, signifies the absence of bubbles, directly visually confirming the suppression of HER during plating. As the potential is swept further to a point closed to maximum deposition (Figure 3c), a dense zinc layer is clearly visible, bubble‐free on both the top and edges of the electrode. During the anodic sweep, Figure 3d shows that the initial stripping has already began and the deposit dissolves evenly and by the start of the next cycle (Figure 3e) the microelectrode is almost devoid of any zinc. This enhanced performance extends consistently across subsequent cycles (Figure 3e–l and Movie 2), demonstrating excellent reproducibility. Each pristine state at the beginning of the respective cycles (Figure 3e,i) confirms that the stripping phase effectively resets the electrode surface, leaving almost no residual zinc. Furthermore, each plating phase (Figure 3f–g,j–k) remains entirely bubble‐free, and each initial stripping phase (Figure 3h,l) proceeds with high uniformity. It should be noted presence of some bubbles are evident during the stripping phase (Movie 2). Overall, the presence of BA facilitates a markedly improved highly reversible plating and stripping process.

The CV curves (Figure S3, Supporting Information), recorded during the in‐situ experiments provide a broader quantitative context for these visual observations. When correlating the dynamic morphological changes observed via LPTEM with the corresponding electrochemical data (Figure S3), it is critical to distinguish between the local visual field and the global electrode response. The in‐situ STEM field of view (FOV) captures a localized area of approximately 7 × 10 µm^2^, whereas the total Pt working electrode spans roughly 7 × 200 µm^2^. The recorded cyclic voltammograms represent the integrated global current across the entire electrode, which is heavily influenced by macroscopic heterogeneities, such as unequal distribution of flow‐induced or HER‐induced gas bubbles. Therefore, the LPTEM images strictly depict localized physical processes, providing direct visual confirmation of mechanisms that cannot be directly correlated with global electrochemical measurements. The most striking quantitative difference is the profound reduction in the maximum cathodic current in the BA‐containing electrolyte reaching approximately—30 µA, compared to roughly ‐140 µA in the base electrolyte at – 2.0 V_vs. Pt_. This excess cathodic current for the base electrolyte is primarily related to the parasitic HER across the complete microelectrode. Furthermore, a distinct shift in the anodic stripping behavior is observed. In the BA‐containing electrolyte, zinc stripping occurs between ∼ ‐1.3 V_vs. Pt_ and ‐1.0 V_vs. Pt_. In stark contrast, the anodic peak for the base electrolyte is significantly delayed, appearing between – 0.75 V_vs. Pt_ and – 0.45 V_vs. Pt_. This substantial shift to more positive potentials indicates that a much higher overpotential is required to strip the zinc deposit in the base electrolyte. This can be linked to the physical presence of the hydrogen bubbles observed in the LPTEM videos, which block the electrochemically active surface area and impose a severe barrier to stripping. Conversely, the bubble‐free interface promoted by BA ensures the electrode remains fully accessible, allowing highly reversible stripping at lower overpotentials.

Having established the general plating and stripping behavior with and without BA, to quantitatively compare the plating behavior we focused on the edge of the electrode. While the absolute potential values obtained using the on‐chip Pt pseudo‐reference electrode cannot be directly translated to a macroscopic bulk cell, applying identical potentials across both electrolytes enables a relative comparison of the deposition dynamics. For this, separate potentiostatic plating experiments were conducted by holding the potential at ‐1.5 V versus Pt. Figure S4 (and Movie 3, Supporting Information) and Figure S5 (and Movie S4, Supporting Information) shows the edge‐plating behavior in the absence and presence of BA, respectively. In the base electrolyte (Figure S4), as before, hydrogen bubbles are clearly visible as dynamic, dark horizontal streaks shifting across the field of view from 12 to 50 s (Figure S4a–f), severely restricting uniform plating and producing a localized, uneven plating morphology. Conversely, in the presence of the BA additive (Figure S5), HER suppression is easily noticeable, econtinuous, more uniform, and denser zinc plating along the entire high‐current edge. Over the 20‐s observation window (Figure S5a–f), the environment remains completely free of bubble‐induced contrast artifacts. This bubble free environment enables zinc to plate as tightly packed, well‐defined faceted structures. To quantitatively capture this visual disparity, Figure S6 (Supporting Information) compares the total deposited area over time for both conditions. The data reveal a significantly faster and more consistent growth rate in the BA‐containing electrolyte, whereas growth in the base electrolyte is severely inhibited due to electrode blockage by gas bubbles.

To probe the suppression of HER during extended plating, a separate experiment was conducted using potentiostatic plating followed by stripping in the presence of BA (Figure S7). During the plating phase, no bubbles were seen during this entire 50‐second plating (Figure S7a–h). Furthermore, subsequent stripping was bubble‐free (Figure S7i–l), leading to almost complete removal of deposited zinc. The complete plating and stripping process can be seen in Movie 5 (Supporting Information).

These in‐situ experiments demonstrate that BA achieves two crucial functions at the nanoscale: it effectively suppresses HER during plating and promotes an improved, highly reversible electroplating process. While the quantitative values from the LPTEM nanoreactor (e.g., potentials and currents) are not directly scalable to the macro‐cell, due to the necessary use of 1 M KOH, a pseudo‐reference electrode, and geometry of the LPTEM nanoreactor, Pt versus Zn substrate, they provide an unambiguous validation of the core functions. These nanoscale visualization forms the basis for the macroscale experiments discussed in the next sections.

### 3.2 Macroscale Consequence I: Compact, Dendrite‐Free Morphology

The in‐situ LPTEM experiments revealed at the nanoscale that BA promotes bubble‐free uniform plating. This section demonstrates how that nanoscale mechanism correlates to the macroscale plating morphology and topography, which was investigated using scanning electron microscopy (SEM) and LSM. For these macroscale tests, zinc was plated at a current density of 10 mA/cm^2^ for 1 h on a mirror‐polished zinc surface in 6 M KOH with 0.2 M ZnO, both with and without BA additives. 0.2 M ZnO was added to the electrolyte to facilitate faster zinc plating and minimize the parasitic HER, consistent with standard macro‐cell testing.

Figures 4a and 4b show that zinc plating in the base 6 M KOH electrolyte exhibits a characteristic leafy, dendritic structure. This morphology, typical for zinc plating in alkaline media [26, 27, 28], is prone to detaching from anode leading to a material loss and short‐circuiting. In stark contrast, the addition of BA to the electrolyte resulted in a complete morphological transformation. The zinc deposits formed in the presence of BA (Figure 4c–h) exhibited a compact, boulder‐like structure. As noted by Zhang et al. [29], such boulder‐like deposits are highly desirable as their compactness minimizes the possibility of dendrite formation and their reduced surface area decreases the corrosion rate. The compact morphology observed was consistent across all tested concentrations of BA: 65 mM (Figure 4c,d), 130 mM (Figure 4e,f), and 245 mM (Figure 4g,h).

The 3D surface profiles from LSM (Figure 4i–l) provide a clear validation of the SEM observations. As LSM primarily scans the topography of the top‐most surface, the resulting images complement the SEM data. In the base electrolyte (Figure 4i), the LSM profile shows a highly nonuniform surface populated by sharp peaks reaching heights of up to 200 µm, which can be linked to the dendritic structures seen in the SEM. Conversely, the BA‐containing electrolytes (Figure 4j–l) appear much flatter. This flatness is not an indication of less plating, but rather the absence of elongated, needle‐like dendrites. This can be corroborated the compact, boulder‐like morphology observed in the SEM images (Figure 4c–h), where the top‐most surface appears considerably more homogeneous. While a direct quantitative comparison of surface roughness can be misleading without complex electric field modeling, the qualitative correlation is unambiguous. Among the concentrations tested, 130 mM BA (Figure 4k) visibly produced the most homogeneous plating, emphasizing its selection as the optimal concentration.

To determine if BA is incorporated into the deposit itself, the crystallographic nature of the deposited zinc was characterized by XRD (Figure S8). The XRD patterns of zinc deposited with and without the BA additive are indistinguishable. Both show prominent reflections for hexagonal close‐packed metallic zinc. The absence of any peak shifts, additional phases, or changes in texture confirms that BA is not incorporated into the zinc lattice. While the absence of bulk peak shifts or secondary crystalline phases suggests that BA does not fundamentally alter the macroscopic zinc crystal lattice, it is important to note that XRD is inherently a bulk‐averaging technique. Consequently, it remains insensitive to subtle, highly localized structural variations that the BA additive might induce at the nanoscale. Capturing these local structural nuances requires high‐resolution techniques such as 4D STEM. However, performing in‐situ 4D STEM currently poses a severe challenge, as the high electron doses required for diffraction mapping rapidly induce beam‐damage and alter the zinc deposits in the highly reactive alkaline liquid environment. To resolve this in future studies, the LPTEM community must pivot toward controlled liquid‐exchange workflows where the reactive electrolyte is carefully flushed and replaced with an inert medium immediately post‐deposition to nanoscale crystallography study.

### 3.3 Macroscale Consequence II: Corrosion and HER Suppression

In addition to improving the deposition morphology, the in‐situ LPTEM experiments provided direct visual evidence that BA suppresses hydrogen bubble formation. The following section presents a quantitative macroscale validation of this behavior, indicating that BA significantly mitigates corrosion and HER in 6 M KOH. Potentiodynamic polarization experiments were conducted to quantify the effect of BA on the corrosion behavior of zinc in a 6 M KOH electrolyte. All experiments were performed at a scan rate of 20 mV/s over a potential range of ‐1.7 V_vs. Hg/HgO_ to ‐1.1 V_vs. Hg/HgO_.

Figure 5a presents the results of the potentiodynamic polarization experiments conducted both in the absence of BA and with varying concentrations of BA. Table 1 provides the corrosion potential (*E*_corr_) and corrosion current density (*I*_corr_) values, derived from Tafel plots within ± 50 mV of the *E*_corr_. The *E*_corr_ of Zn in 6 M KOH was found to be approximately ‐1.47 V_vs Hg/HgO_. Figure 5a shows that the addition of BA to the electrolyte mainly affected the cathodic process, leading to a shift in the corrosion potential towards more negative values. Notably, increasing the BA concentration shifted the potential from ‐1.47 V_vs Hg/HgO_ to ‐1.51 V_vs Hg/HgO_ at 245 mM BA in 6 M KOH. A lower *E*_corr_ indicates that the HER occurs at more negative potentials, thereby increasing the charging efficiency [30]. Wu et al. [14] also observed an increase in HER overpotential with the addition of BA in lead‐acid battery electrolytes, while Karwas et al. [15] reported a reduction in HER and enhanced metal plating efficiency when BA was added to electroplating baths. Thus, the results obtained in this study suggest that BA suppresses HER, which has a potential to increase charging efficiency. Moreover, a significant decrease in corrosion current was observed with the addition of BA. As shown in Table 1, the introduction of 130 mM BA reduces the corrosion current density by nearly an order of magnitude (from 349 to 36 µA/cm^2^). Consequently, the calculated electrochemical corrosion rate drops from 8 to 0.85 mg/cm^2^/day. Although a further increase in BA concentration to 245 mM provides only a slight additional improvement, the results indicate that 130 mM BA represents the most effective concentration for corrosion inhibition under the investigated conditions. To investigate the time‐dependent influence of BA on the electrochemical behavior of Zn, potentiodynamic polarization measurements were repeated after immersing the zinc electrode in the 130 mM BA electrolyte for 30 min, allowing potential surface adsorption processes to further develop. As shown in Figure 5b and Table S1 (Supporting Information), the extended equilibration period resulted in an additional reduction of the corrosion current density by approximately 30% compared to the 5‐min measurement, suggesting the gradual establishment of a relatively stable adsorbed layer at the zinc/electrolyte interface.

**TABLE 1:** The influence of the boric acid concentration on the corrosion behavior of Zn electrodes. *E*_corr_, *I*_corr_, and electrochemical corrosion rates are obtained from the potentiodynamic polarization curves (Figure 5a). Gravimetric weight loss of the Zn electrodes is also provided upon 24 h of immersion.

Electrolyte	*E*_corr_ (V_vs Hg/HgO_)	*I*_corr_ (µA/cm^2^)	Electrochemical corrosion rate (mg/cm^2^/day)	Weight loss (mg/cm^2^/day)
6 M KOH	−1.47 ± 0.006	349 ± 31	8 ± 0.7	0.33 ± 0.06
6 M KOH + 65 mM BA	−1.49 ± 0.016	190 ± 82	4.5 ± 1.4	0.44 ± 0.02
6 M KOH + 130 mM BA	−1.50 ± 0.006	36 ± 6.6	0.85 ± 0.14	0.16 ± 0.0156
6 M KOH + 245 mM BA	−1.516 ± 0.006	30 ± 6.5	0.66 ± 0.07	0.24 ± 0.04

Since zinc corrosion in aqueous alkaline electrolytes represents a significant challenge [31, 32], further corrosion analysis was performed through gravimetric weight‐loss measurements of Zn electrodes. To evaluate this behavior, polished zinc discs were immersed in electrolytes with and without BA additives, and their mass was recorded after one day of immersion (see Table 1). The results reveal a clear dependence of the corrosion behavior on the BA concentration, with the electrolyte containing 130 mM BA exhibiting the lowest weight loss among the investigated conditions. This suggests that an optimum BA concentration improves the long‐term corrosion resistance of Zn under open‐circuit conditions. Such non‐monotonic additive effects have also been reported for Zn in concentrated KOH, where intermediate concentrations of PEG‐, surfactant‐, or polymer‐based additives provided the most favorable balance between corrosion inhibition, passivation, and interfacial transport [11, 33, 34, 35]. Moreover, it is noteworthy to mention that the Tafel‐derived *I*_corr_ and 24 h gravimetric measurements should be regarded as complementary probes of different stages of the evolving Zn/electrolyte interface rather than as quantities expected to exhibit identical concentration dependence.

The effect of BA on zinc electrochemistry was further evaluated during galvanostatic stripping through gravimetric weight‐loss measurements performed after stripping experiments at a current density of 10 mA cm^−^^2^ over a period of 24 h. The weight loss was determined by comparing the electrode mass before and after the stripping process. The theoretical weight loss for the stripping process, calculated based on the theoretical specific capacity of zinc, was determined to be 0.3705 g. Experimental measurements revealed that the weight loss of zinc electrodes stripped in bare 6 M KOH exceeded the theoretical value, indicating the occurrence of additional zinc consumption through parasitic corrosion processes during electrochemical operation. Under these conditions, approximately 3.5% of the zinc loss could be attributed to corrosion. In comparison, the incorporation of 130 mM BA as an electrolyte additive substantially reduced the excess material loss, with only ∼0.6% of the zinc estimated to be consumed by corrosion during the same stripping process. Detailed results can be found in Table S2. These results further support the beneficial role of BA in mitigating parasitic zinc dissolution and improving zinc utilization efficiency.

To further substantiate the suppression of HER observed visually in the nanoreactor, quantitative macroscale gas evolution measurements were performed utilizing a practical Zn‐anode [25] during the first charging cycle. As shown in Table S3, the incorporation of 130 mM BA into the 6 M KOH + 0.2 M ZnO electrolyte resulted in a > 30% reduction in hydrogen gas evolved compared to the base electrolyte. This reduction in HER, which aligns directly with the bubble‐free electrode interface visualized during the in‐situ LPTEM experiments, further confirms that BA actively mitigates HER in practical cell environments.

To further examine the influence of BA on Zn redox behavior, cyclic voltammetry measurements were performed in the investigated electrolytes (Figure S9). In 6 M KOH, a cathodic feature centered at approximately −1.47 V versus Hg/HgO is observed and is attributed predominantly to the reduction of soluble zincate species and Zn electrodeposition. Upon addition of BA, the cathodic peak becomes moderately larger in magnitude, while the subsequent anodic current associated with oxidation/dissolution of Zn also increases. The cathodic peak potential remains broadly similar, with only a small shift upon BA addition. These results indicate that BA modifies the Zn deposition/dissolution response and is consistent with enhanced electrochemical utilization of Zn species under the investigated conditions. However, because the measured cathodic current may contain contributions from both Zn deposition and the competing HER, the CV data alone cannot quantitatively establish an increase in Zn‐plating Faradaic efficiency or prove HER suppression.

To assess the chemical stability of BA under operating conditions, the boron concentration in the electrolyte was monitored by inductively coupled plasma optical emission spectrometry (ICP‐OES) during stripping and plating in Zn||Zn symmetrical cells (Table 2). The boron concentration remained essentially unchanged throughout cycling, demonstrating that BA is not consumed during the electrochemical process and remains dissolved in the electrolyte. The ICP‐OES results indicate that BA‐derived boron is retained in the electrolyte and is not significantly depleted during cycling.

**TABLE 2:** ICP results showing amount of different element present at the electrolyte at different charge‐discharge states in Zn||Zn cell.

	Amount in g/l		
	Boron	Potassium	Zinc
Initial state	1.35 ± 0.04	236 ± 9	12.8 ± 0.5
After 30 min charging	1.35 ± 0.08	236 ± 11	12.4 ± 0.6
After 1 h charging	1.46 ± 0.07	237 ± 12	12.9 ± 0.2
After 30 min discharging	1.46 ± 0.08	230 ± 13	13.2 ± 0.7
After 1 h discharging	1.39 ± 0.03	239 ± 8	14 ± 0.4

In this study, a multimodal workflow was employed to bridge disparate analytical scales. In‐situ TEM was utilized specifically to understand the dynamic effects of the electrolyte additive at the nanoscale, such as the suppression of gas bubbles and reversibility. However, due to the restricted electrolyte concentrations required for LPTEM and the geometric differences from lab‐scale macro cells, the definitive morphology and topographical uniformity of the deposits were characterized using ex‐situ SEM and LSM. Enforcing a strict link between in‐situ nanoscale observations and macroscopic behavior is essential for the future of battery research. However, the LPTEM community must ultimately transition to monolithic nanochannel chips to enable in‐situ EELS to understand detailed plating and stripping mechanisms, despite the current engineering challenges of managing HER‐induced bubbles in ∼50 nm channels. Finally, to demonstrate the applicability of BA in a practical alkaline system, we have cycled our recently developed ZnO‐PTFE electrode with 2 wt.% Bi_2_O_3_ electrode additive [25] in 6 M KOH+0.2 ZnO+130 mM BA at a currently of 120 mAh/g_Zn_ at 30% DOD. Figure S10 shows the charge and discharge capacity and the resultant coulombic efficiency over 75 cycles. The steady 100% coulombic efficiency achieved during 75 cycles demonstrate the potential of BA as a practical electrolyte additive and now awaits full cell testing.

## 4 Conclusion

In this study, a systematic, multimodal workflow was established to bridge fundamental nanoscale observations with practical macroscale battery reality. First, in‐situ LPTEM in 1 M KOH directly visualized the interfacial processes at the nanoscale, proving that the boric acid additive strongly suppresses parasitic hydrogen evolution, which leads to bubble‐free, uniform zinc plating and highly reversible stripping. Next, to understand the macroscopic implications in a standard 6 M KOH battery environment, ex‐situ SEM and LSM were coupled with bulk electrochemical measurements. This correlation revealed that the addition of BA transforms the dendritic zinc deposit morphology into a desirable, compact, boulder‐like structure. By combining these structural analyses with potentiodynamic testing, 130 mM BA was identified as the optimal concentration, acting as a potent corrosion inhibitor that reduces the corrosion current density by nearly an order of magnitude (from 349 to 36 µA/cm^2^). Crucially, comprehensive bulk electrolyte characterization and structural analyses of the deposits, combining XRD and ICP‐OES, suggest that in the electrolyte, BA is not consumed during cycling and functions as a non‐consumed surface‐active agent—it likely gets adsorbed onto the zinc surface to promote uniform nucleation and suppress HER. Finally, to validate the real‐world efficacy of this optimized electrolyte, it was applied to a practical ZnO‐PTFE composite anode. The resulting cell delivered over 75 cycles at 100% coulombic efficiency, proving that the additive operates effectively under realistic conditions. This work not only introduces a simple and effective additive for alkaline zinc batteries but also showcases a robust roadmap from in‐situ nanoscale visualization to realistic macroscale optimization and long‐term cycling to forge the crucial link between fundamental observation and the development of practical, high‐performance energy storage systems.

## Author Contributions

**Sarmila Dutta**: conceptualization, investigation, Writing – original draft, methodology, validation, visualization, Writing – review and editing, formal analysis, data curation. **Yasin Emre Durmus**: supervision, writing – review and editing, project administration. **Eunmi Im**: data curation, writing – review and editing. **Hongyu Sun**: resources. **Hugo Pérez Garza**: resources. **Shibabrata Basak**: supervision, writing – review and editing. **Hans Kungl**: writing – review and editing. **Hermann Tempel**: writing – review and editing, project administration. **Rüdiger‐A. Eichel**: funding acquisition, writing – review and editing, project administration, supervision.

## Conflicts of Interest

The authors declare no conflicts of interest.

## Supporting information




**Supporting File 1**: smtd71069‐sup‐0001‐Movie1.avi.

**Supporting File 2**: smtd71069‐sup‐0002‐Movie2.avi.

**Supporting File 3**: smtd71069‐sup‐0003‐Movie3.avi.

**Supporting File 4**: smtd71069‐sup‐0004‐Movie4.avi.

**Supporting File 5**: smtd71069‐sup‐0005‐Movie5.avi.

**Supporting File 6**: smtd71069‐sup‐0006‐SuppMat.docx.

## Data Availability

The data that supports the findings of this study are available in the Supplementary Material of this article.
