# Supplementary material for: A Multimodal In‐Situ Liquid Phase TEM and Electrochemical Study on Boric Acid as Electrolyte Additive for Alkaline Zinc‐Air Batteries
Source: Small Methods. 2026 Oct 12;10(21):e71069. doi: 10.1002/smtd.71069 (PMC13745979; doi:10.1002/smtd.71069)
Supplement: Supplementary file 1 [file SMTD-10-e71069-s002.docx]

**Supplementary Information**

**A multimodal in-situ LPTEM study on boric acid as electrolyte additive for alkaline zinc-air batteries**

Sarmila Dutta^a,b^, Yasin Emre Durmus^a, *^, Eunmi Im^a^, Hongyu Sun^c^, Hugo Pérez Garza^c^, Shibabrata Basak^a, *^, Hans Kungl^a^, Hermann Tempel^a^, Rüdiger-A. Eichel ^a, b, d^

^a^ Institute of Energy Technologies - Fundamental Electrochemistry (IET-1), Forschungszentrum Jülich GmbH, Jülich, Germany

**^b^ Institute of Physical Chemistry, RWTH Aachen University, Aachen, Germany**

**^c^ DENSsolutions B.V., Delft, Netherlands**

**^d^ Faculty of Mechanical Engineering, RWTH Aachen University, Aachen, Germany**

*** Corresponding Authors:** [y.durmus@fz-juelich.de](mailto:y.durmus@fz-juelich.de) **(Y. E. Durmus)** [s.basak@fz-juelich.de](mailto:s.basak@fz-juelich.de) **(S. Basak)**

**Figure S1. Illustrates the in situ TEM holder. (a) Photograph of in situ liquid phase TEM holder highlighting the liquid inlet/outlet ports, biasing cable port and tip of the holder. (b) Photograph of the tip of the holder with only the bottom MEMS chip. The bottom chip consists of Pt working, counter, and reference electrodes. In the magnified image on the right side working (WE), counter (CE) and reference electrode (RE) can be seen. (c) Schematic representation of the liquid cell. The on-chip flow (not shown) allows liquid insertion within the sandwich MEMS chips.**

**Figure S2.** STEM snapshot of the Pt microelectrode at OCV in BA containing electrolyte before the electrochemical cycling.

**Figure S3. Shows the CV plots with and without BA as electrolyte additive at a scan rate of 0.1 V/s in the MEMS nanoreactor. Electrolyte flow rate during the experiments was 1.3 µL/min.**

**Figure S4. HAADF STEM images during plating at -1.5V_vs Pt_ in absence of BA. Left top corner indicate the time passed during the experiment in mm:ss format.**

**Figure S5. HAADF STEM images during plating at -1.5V_vs Pt_ in presence of BA. Left top corner indicate the time passed during the experiment in mm:ss format.**

**Figure S6. Compares the deposited area during plating at -1.5Vvs Pt in base electrolyte and in** 1 M KOH+30 mM ZnO+0.1 wt.% BA. The area was calculated by manual segmentation utilizing ImageJ.

**Figure S7.** HAADF STEM images during plating and stripping in the base + BA electrolyte. a)-h) snap shots during plating; i)-l) snap shots of during stripping. Left top corner indicate the time passed during the experiment in mm:ss format.

**Figure S8.** XRD patterns of Zn deposited at a current density of 10 mA/cm² with and without 130mM BA.

**Figure S9.** Cyclic voltammetry in 6M KOH with and without 130mM BA between -1.3 V to -1.7 V vs. Hg/HgO at a scan rate of 5 mV/s.

**Figure S10.** Cycling of ZnO-PTFE electrode with 2 wt.% Bi_2_O_3_ electrode additive in 6M KOH electrolyte with 0.2M ZnO and 130mM boric acid at a current density of 120mAh/g_Zn_ at 30% DOD.

**Table S1.** Shows the Ecorr, Icorr values and electrochemical corrosion rates of zinc in 6 M KOH +0.6 wt.% BA electrolyte after 5 and 30 mins of immersion, obtained from the Potentiodynamic Polarization curves (Figure 3b).

| **Immersion time** | **E_corr_ (V_vs Hg/HgO_)** | **I_corr_ (μA/cm^2^)** | **Electrochemical corrosion rate (mg/cm^2^/day)** |
| --- | --- | --- | --- |
| 5 min | -1.50±0.006 | 36 ± 6.6 | 0.85 ± 0.14 |
| 30 min | -1.514±0.0014 | 22 ± 2 | 0.52 ± 0.05 |

**Table S1.** Shows the weight loss of zinc electrode in 6 M KOH electrolyte with and without boric acid additive over a period of 24 hours during stripping process at a current density of current density of 10 mA/cm².

| **Electrolyte** | **Total Weight loss (mg/cm^2^/day)** | **Consumption due to corrosion (%)** |
| --- | --- | --- |
| 6 M KOH | 0.3838±0.00035 | 3.5 |
| 6 M KOH+0.3 wt.% BA | 0.377 ± 0.006 | 0.6 |
| 6 M KOH+0.6 wt.% BA | 0.373 ± 0.007 | 0.6 |
| 6 M KOH+1.2 wt.% BA | 0.373 ± 0.005 | 0.6 |

**Table S3.** Compares average hydrogen evolution during charging using ZnO-PTFE electrode with and without electrolyte additives.

| **Anode** | **Electrolyte** | **Average value of H_2_ ( in ppm) during 1hr of charging at 60mA/g_Zn_** |
| --- | --- | --- |
| ZnO -PTFE+ 2%Bi_2_O_3_ | 6 M KOH | 1792 |
| ZnO -PTFE+ 2%Bi_2_O_3_ | 6 M KOH + 0.2 M ZnO | 1767 |
| ZnO -PTFE + 2%Bi_2_O_3_ | 6 M KOH + 0.2 M ZnO + 130mM BA | 1152 |

**Movie 1.** Shows the HAADF STEM movie during the plating and stripping process during the five CV cycles in the nanoreactor in the base electrolyte, i.e., in 1 M KOH+30 mM ZnO.

**Movie 2.** Shows the HAADF STEM movie during the plating and stripping process during five CV in the nanoreactor in the 1 M KOH+30 mM ZnO+0.1 wt.% BA.

**Movie 3.** Shows the HAADF STEM movie during the plating at -1.5 V_vs Pt_ in 1 M KOH+30 mM ZnO+0.1 wt.% BA.

**Movie 4.** Shows the HAADF STEM movie during the plating at -1.5 V_vs Pt_ in the base electrolyte, i.e., in 1 M KOH+30 mM ZnO.

**Movie 5.** Shows the HAADF STEM movie during the plating and stripping process during five CV cycles in the nanoreactor in the 1 M KOH+30 mM ZnO+0.1 wt.% BA. In order to see the effect of more plating, CV was paused at -1.5 V for 35 s before stripping.
